# Hepatic veno-occlusive disease/sinusoidal obstruction syndrome after hematopoietic stem cell transplantation for thalassemia major: incidence, management, and outcome

**DOI:** 10.1038/s41409-021-01233-w

**Published:** 2021-02-19

**Authors:** Xiaoxuan Lai, Lianjin Liu, Zhongming Zhang, Lingling Shi, Gaohui Yang, Meiqing Wu, Rui Huang, Rongrong Liu, Yongrong Lai, Qiaochuan Li

**Affiliations:** Department of Hematology, The First Affiliated Hospital of Guangxi Medical University, Nanning, Guangxi China

**Keywords:** Combination drug therapy, Anaemia, Risk factors

## Abstract

Hepatic veno-occlusive disease or sinusoidal obstruction syndrome (VOD/SOS) is a potentially life-threatening complication of allogeneic hematopoietic stem cell transplantation (allo-HSCT). In the present prospective study, we aimed to investigate the incidence, management, and outcome of VOD/SOS in patients with thalassemia major (TM) who received allo-HSCT. VOD/SOS was diagnosed and classified based on the modified Seattle criteria. The prophylactic regimen for VOD/SOS was a combination treatment of dalteparin and lipo-PGE1. VOD/SOS was managed through an approach consisting of adequate supportive measures, short-term withdrawal of calcineurin inhibitors (CNIs), and the use of methylprednisolone and basiliximab for graft-versus-host disease prophylaxis. VOD/SOS was found in 54 of 521 patients (10.4%) at a median time of 12 days after allo-HSCT. The cumulative incidence of all-grade and moderate VOD/SOS was 10.4% and 4.2%, respectively. Among the 54 VOD/SOS patients, no patient developed severe grade and died from VOD/SOS. Besides, the cumulative incidence of transplant-related mortality on day 100 for patients with or without VOD/SOS was 0% vs. 4.0% (*P* = 0.187), respectively, and the 3-year overall survival rates were 94.3% vs. 93.2% (*P* = 0.707), respectively. Collectively, we concluded that appropriate symptomatic therapy and short-term withdrawal of CNIs safely mitigated the mortality of VOD/SOS in TM patients who underwent allo-HSCT.

## Introduction

Hepatic veno-occlusive disease/sinusoidal obstruction syndrome (VOD/SOS) is a potentially fatal complication of hematopoietic stem cell transplantation (HSCT). The VOD/SOS primarily damages both sinusoidal endothelial cells and hepatocytes in zone 3 of the hepatic acinus, and such process is triggered by several factors, such as the toxicity of the conditioning regimen, the release of cytokines due to inflammation and engraftment, and graft-versus-host disease (GvHD) prophylactic regimen [1–3]. The risk factors include thalassemia major (TM), very young or old age, ferritin levels, a history of previous liver disease, conditioning regimen consisting of busulfan (Bu) and cyclophosphamide (Cy), and the use of calcineurin inhibitors (CNIs) for GvHD prophylaxis [4]. The only proved curative therapy of VOD/SOS is defibrotide [5].

The only established curative option for TM is allogeneic HSCT (allo-HSCT). It is expected that more than 90% of TM patients can survive after allo-HSCT with a thalassemia-free survival (TFS) of around 80% [6]. However, the VOD/SOS is a common complication of TM patients after allo-HSCT, which can probably be attributed to the pre-existing liver damage caused by iron overload, conditioning regimen consisting of Bu and Cy, and the use of CNIs for GvHD prophylaxis. In this prospective study, we reported the incidence, management, and outcome of VOD/SOS in 521 TM patients who underwent allo-HSCT in our center.

## Patients and methods

### Patients

A total of 521 TM patients were enrolled to assess the VOD/SOS in the present study (Table 1) between July 2007 and July 2019. These patients received HLA-matched sibling donor (MSD) transplants, unrelated donor (URD) transplants, and haploidentical transplants. This study was approved by the local institutional review board, and the outcome data were reported to the Chinese Bone Marrow Transplant Registry (CBMTR). Written informed consent was obtained from all the parents of the patients following the Declaration of Helsinki. All patients had a Lansky or Karnofsky performance score ≥90%. The baseline values of bilirubin, liver size, and body weight before transplantation were determined. The patient characteristics were summarized in Table 1.

**Table 1:** Main demographic and transplant data of the patients.

Characteristics	No-VOD (*N* = 467)	VOD (*N* = 54)	*P* value
Age-yr	5 (2–19)	5 (2–12)	0.256
Male/sex-no. (%)	300 (64.2)	35 (64.8)	0.400
Liver size >5 cm-no. (%)	65 (13.9)	6 (11.1)	0.569
Bilirubin (mg/dL)	0.7 (0.2–2.5)	0.6 (0.3–2.3)	0.876
Median serum ferritin (ng/mL)	3095 (465–10,336)	3558 (624–11,473)	0.425
Pesaro classification			0.907
Class I-no. (%)	72 (15.4)	8 (14.8)	
≥Class II-no. (%)	395 (84.6)	46 (85.2)	
Donor: female for male-no. (%)	148 (31.7)	20 (37.0)	0.993
Donor type			0.433
MSD-no. (%)	357 (76.4)	37 (68.5)	
URD-no. (%)	89 (19.1)	14 (25.9)	
Haplotype donor-no. (%)	21 (4.5)	3 (5.6)	
Cell source			0.310
PBSC-no. (%)	90 (19.3)	14 (25.9)	
BM-no. (%)	15 (3.2)	0	
CB-no. (%)	8 (1.7)	1 (1.9)	
CB + BM-no. (%)	158 (33.8)	22 (42.7)	
PBSC + BM-no. (%)	196 (42.0)	17 (31.5)	
ABO incompatibility-no. (%)	178 (38.1)	26 (48.1)	0.153
Infused MNC-10^8^/kg	12.4 (0.5–35.4)	14.3 (0.5–31.2)	0.699
Infused CD34^+^ cells-10^6^/kg	7.5 (0.3–32.4)	8.1 (0.2–32.9)	0.519
Sepsis post-HSCT-no. (%)	64 (13.7)	4 (7.4)	0.193
No. of platelet transfusions	4 (0–26)	6 (1–23)	0.005
No. of platelet transfusions over 12 days	3 (0–10)	4 (1–11)	0.096
Platelet refractoriness-no. (%)	68 (14.6)	9 (16.7)	0.680
Days to ANC >0.5 × 10^9^/L	11 (7–32)	12 (8–22)	0.585
Days to PC >20 × 10^9^/L	14 (9–49)	17 (9–47)	0.010
Acute GvHD			0.129
Grade I-no. (%)	23 (4.9)	6 (11.1)	
Grade II-no. (%)	50 (10.7)	7 (13.0)	
Grade III-no. (%)	21 (4.5)	1 (1.9)	
Grade IV-no. (%)	10 (2.1)	3 (5.6)	
Chronic GvHD			0.896
Mild-no. (%)	13 (2.7)	2 (3.8)	
Moderate-severe-no. (%)	11 (2.4)	1 (1.9)	

*ANC* absolute neutrophil count, *BM* bone marrow, *CB* cord blood, *GvHD* graft-versus-host disease, *HSCT* hematopoietic stem cell transplantation, *MNC* mononuclear cell, *MSD* HLA-matched sibling donor, *PBSC* peripheral blood stem cell, *PC* platelet count, *URD* unrelated donor, *VOD* hepatic veno-occlusive disease.

### Conditioning regimen and GvHD prophylaxis

The conditioning regimen consisted of Bu, Cy, fludarabine (Flu), and anti-thymocyte globulin (ATG). The detailed regimen was as follows: (1) Bu (1 mg/kg) was intravenously (IV) administered four times per day for 4 days (day −9 to day −6); (2) Flu (50 mg/m^2^/day) was IV administered for 3 days (day −12 to day −10); (3) Cy (50 mg/kg/day) was IV administered for 4 days (day −5 to day −2); and (4) ATG (thymoglobulin, 2.5 mg/kg/day) was IV given for 4 days (days −4 to day −1) [7]. GvHD prophylactic regimen for MSD HSCT consisted of cyclosporine A (CsA), methotrexate (MTX), and mycophenolate mofetil (MMF) [8]. GvHD prophylactic regimen for URD HSCT and haploidentical HSCT consisted of tacrolimus, MTX, and MMF.

### Diagnosis and classification of VOD/SOS

VOD/SOS could be diagnosed when two of the following clinical findings presented within 30 days after HSCT according to the modified Seattle criteria [9, 10]: (1) hyperbilirubinemia more than 2 mg/dL; (2) ascites (radiographic examination) and/or unexplained weight gain (2% above baseline weight); and (3) hepatomegaly over baseline or pain in the right upper quadrant. The severity of VOD/SOS was defined according to established criteria as follows: mild for clinically manifested VOD/SOS that was resolved without intervention; moderate for VOD/SOS that required treatment but was resolved completely; and severe for VOD/SOS that caused death or progressed to multi-organ failure (MOF). MOF was defined as either an oxygen requirement with an oxygen saturation of <90% on room air and/or ventilator dependence; renal insufficiency (doubling of baseline creatinine level and/or dialysis dependence); and/or encephalopathy [1, 10, 11].

### Prophylaxis and management of VOD/SOS

The prophylactic regimen for VOD/SOS was a combination treatment of dalteparin and lipo-PGE1. Patients were subcutaneously administered with dalteparin at a dose of 100 IU/kg/day. Lipo-PGE1 was IV infused at a dose of 1 μg/kg/day. Prophylactic therapy consisting of dalteparin and lipo-PGE1 was given until day 21. Once VOD/SOS was clinically diagnosed, standard supportive care measures were adopted, such as the restriction of daily sodium and fluid intake, diuretics, and hematologic support. All patients diagnosed with VOD/SOS were timely administered with dalteparin at a dose of 100 IU/kg, twice daily. CNIs were immediately discontinued for all patients diagnosed with VOD/SOS. The methylprednisolone and anti-CD25 monoclonal antibody (basiliximab) were administered to continue the prophylactic or therapeutic regimen of GvHD. After the clinical symptoms of VOD/SOS were improved, CNIs were resumed to continue the prophylactic or therapeutic regimen of GvHD.

### Definitions

The time to VOD/SOS was calculated from the date of HSCT to the date of clinical diagnosis. Neutrophil engraftment and platelet engraftment were defined as the first three consecutive days when the absolute neutrophil count and an unsupported platelet count were >0.5 × 10^9^/L and >20 × 10^9^/L, respectively. Platelet refractoriness was defined as a corrected count increment of less than 10,000/µL following at least two sequential fresh platelet transfusions. Transplant-related mortality (TRM) was defined as transplantation-related deaths instead of the recurrence of TM. Overall survival (OS) was defined from the date of transplantation to the date of death or last follow-up. TFS was defined from the date of transplantation to either the recurrence of transfusion-dependent thalassemia or the death from any cause. Acute and chronic GvHD were classified by Glucksberg and National Institutes of Health classifications [12, 13]. GvHD-free and relapse-free survival (GRFS) was defined as the absence of relapse, death from any cause, grade 3 to 4 acute GvHD, and chronic GvHD requiring systemic treatment.

### Statistical analyses

The median follow-up time was 38 months, ranging from 1 to 150 months. The primary objective of this study was to determine the cumulative incidence of VOD/SOS and treatment outcome in TM patients. Cumulative incidence estimates were used to determine the incidences of GvHD and VOD/SOS. The probabilities of OS, TFS, and GRFS were evaluated using the Kaplan–Meier method. Results were expressed as a probability or cumulative incidence (%) with a 95% confidence interval (95% CI). Chi-square statistics was used for discrete variables to compare characteristics of patients, donors, and transplants between groups, and the Mann–Whitney test was employed for continuous variables. Both univariate and multivariate analyses of prognostic factors were carried out according to the log-rank test and a stepwise Cox proportional hazards regression model, respectively. The effects of the following parameters on the development of VOD/SOS were examined: (1) patient characteristics (age, sex, ferritin level, liver size, and risk classification for TM patients), (2) donor characteristics (donor type, female/male donor-recipient combination, cell source, and ABO incompatibility), and (3) transplantation-related factors (development of GvHD, sepsis post-HSCT, and platelet refractoriness). All statistical analyses were performed using SPSS 18.0 software (SPSS, Chicago, IL, USA), except for the cumulative incidence analyses, which were conducted using NCSS software (NCSS, Kaysville, UT, USA).

## Results

### Incidence of VOD

Among the 521 transplants, 54 patients were diagnosed with VOD/SOS at a median time of 12 days (range, 2–43) after HSCT. At diagnosis, 68.5% (37/54) of SOS/VOD patients had a bilirubin level <2 mg/dL, and 70.3% (38/54) of VOD/SOS patients had an increased bilirubin level from a baseline value in three consecutive days. Moreover, 41 patients (75.9%) had ascites. All VOD/SOS patients had a weight gain >5% of the baseline value and hepatomegaly of increased size over pre-HSCT. Retrospectively, all VOD/SOS patients in this cohort also met the EBMT diagnostic criteria for hepatic SOS/VOD in children and the modified VOD/SOS diagnostic criteria reported by Cairo et al. [1, 2]. The cumulative incidence of all-grade VOD/SOS and moderate VOD/SOS was 10.4% (95% CI, 8.9–13.4) and 4.2% (95% CI, 2.8–6.4), respectively. The cumulative incidence of moderate VOD/SOS in matched unrelated donor (MUD) HSCT, MSD HSCT, and haploidentical HSCT was 8.7% (95% CI, 4.7–16.3), 3.1% (95% CI, 1.8–5.4), and 4.2% (95% CI, 0.6–28.4), respectively. Moderate VOD/SOS was more frequently detected in MUD HSCT compared with MSD HSCT (*P* = 0.010). No differences in terms of the total number of infused nucleated cells or CD34^+^ cells, as well as neutrophil engraftment, were found between patients with or without VOD/SOS. Platelet engraftment was delayed, and the number of platelet transfusions was higher in VOD/SOS patients (Table 1). Univariate analysis revealed that none of the above-mentioned factors reached significance for all-grade VOD/SOS (Table 2).

**Table 2:** Univariate analysis of risk factors for the development of VOD.

Variables	*N*	VOD % (95% CI)	Hazard ratio (95% CI)	*P* values
Age-yr				
≥7 yr	152	8.6 (5.1–14.4)	1	
>4 and <7 yr	177	11.0 (7.3–16.5)	1.310 (0.656–2.616)	0.444
≤4 yr	192	11.3 (7.5–17.1)	1.354 (0.673–2.721)	0.396
Pesaro classification				
Class I	80	10.1 (5.2–19.5)	1	
≥Class II	441	10.5 (8.0–13.7)	1.016 (0.480–2.153)	0.996
Serum ferritin				
> 3000 ng/mL	270	9.3 (6.4–13.5)	1	
≤ 3000 ng/mL	251	11.6 (8.3–16.4)	1.296 (0.759–2.213)	0.342
Donor type				
MSD	394	9.4 (6.9–12.8)	1	
Haplotype	24	12.5 (4.3–36.0)	1.348 (0.416–4.373)	0.220
URD	104	13.4 (8.4–22.1)	1.469 (0.794–2.717)	0.619
Liver size >5 cm				
Yes	71	8.5 (3.9–18.2)	1	
No	450	10.7 (8.2–14.0)	1.28 (0.548–2.990)	0.569
ABO incompatibility				
No	317	8.9 (6.2–12.7)	1	
Yes	204	12.8 (8.9–18.3)	1.473 (0.864–2.512)	0.155
Sepsis post-HSCT				
Yes	68	6.0 (2.3–15.6)	1	
No	453	11.0 (8.5–14.4)	1.876 (0.678–5.194)	0.226
Platelet refractoriness				
No	444	10.2 (7.7–13.4)	1	
Yes	77	11.7 (6.3–21.6)	1.150 (0.562–2.351)	0.703

*HSCT* hematopoietic stem cell transplantation, *MSD* HLA-matched sibling donor, *N* number of cases, *URD* unrelated donor, *VOD* hepatic veno-occlusive disease.

### Treatment and outcome of VOD/SOS

All VOD/SOS patients received appropriate supportive care, methylprednisolone, and basiliximab for the prophylactic or therapeutic regimen of GvHD, and CNIs were immediately terminated after clinical diagnosis of VOD/SOS. Among 54 VOD/SOS patients, mild and moderate VOD/SOS were found in 32 (59.3%) and 22 (40.7%) cases, respectively. No patient developed severe VOD/SOS. All VOD/SOS patients were resolved after a median of 8 days (range, 6–15). After the clinical symptoms of VOD/SOS were improved, CNIs were resumed to continue the prophylactic or therapeutic regimen of GvHD in all VOD/SOS patients. The median time for short-term withdrawal of CNIs was 8 days, ranging from 7 to 15 days. No patient died from VOD/SOS.

### GvHD

The cumulative incidence of all-grade acute GvHD for patients with or without VOD/SOS was 31.5% (95% CI, 21.2–46.7) vs. 22.6% (95% CI, 19.0–26.7) (*P* = 0.107), respectively, and such value for grade II–IV acute GvHD and grade III–IV acute GvHD was 20.4% (95% CI, 12.0–34.5) vs. 17.8% (95% CI, 14.7–21.7) (*P* = 0.707), and 7.4% (95% CI, 2.9–19.0) vs. 6.7% (95% CI, 4.8–9.5) (*P* = 0.845), respectively. The cumulative incidence of all-grade chronic GvHD for patients with or without VOD/SOS was 6.2% (95% CI, 2.1–18.8) vs. 5.9% (95% CI, 0.4–8.8) (*P* = 0.956), respectively, and such value for moderate-severe chronic GvHD was 2.3% (95% CI, 0.4–16.9) vs. 2.8% (95% CI, 1.6–5.1) (*P* = 0.731), respectively. Eight (8/54, 14.8%) VOD/SOS patients developed acute GvHD after VOD/SOS, and the time for acute GvHD onset was 21 (3–58) days after VOD/SOS. All of the patients with acute GvHD and chronic GvHD favorably responded to immunosuppressive treatment (IST). No one died from GvHD.

### Treatment outcome

**Fig. 1:**
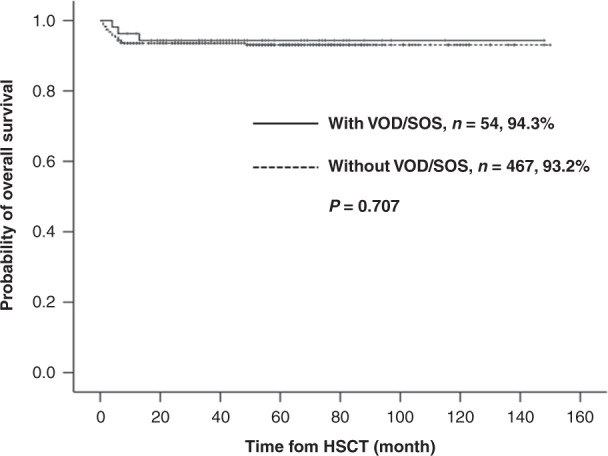
Probability for overall survival of TM with or without VOD/SOS. Kaplan–Meier probability for OS of TM with or without hepatic VOD/SOS.

Three of 54 VOD/SOS patients (5.6%) died after HSCT, including two deaths due to post-transplant lymphoproliferative disorders, and one death was attributed to interstitial pneumonia. No patients died from VOD/SOS. The cumulative incidence of TRM on day 100 for patients with or without VOD/SOS was 0% vs. 4.0% (95% CI, 2.4–6.5) (*P* = 0.187), respectively. The 3-year TRM rates were 5.7% (95% CI, 1.9–17.0) vs. 6.4% (95% CI, 4.5–9.1) (*P* = 0.796), respectively. The 3-year OS rates were 94.3% (95% CI, 88.1–100) vs. 93.2% (95% CI, 90.9–95.5) (*P* = 0.707), respectively, (Fig. 1). The 3-year TFS rates were 92.5% (95% CI, 85.4–99.6) vs. 92.7% (95% CI, 90.3–95.1) (*P* = 0.996), respectively. The 3-year GRFS rates were 86.9% (95% CI, 77.9–96.0) vs. 86.0% (95% CI, 82.8–89.2) (*P* = 0.810), respectively.

## Discussion

The incidence of VOD/SOS mainly depends on the patients’ status, underlying disease, type of HSCT, conditioning regimen, VOD/SOS prophylactic strategy, and the use of different diagnostic criteria [4]. International Blood and Marrow Transplant Research (CIBMTR) has reported that the incidence of VOD/SOS is 4.9% in 13,097 patients receiving allo-HSCT [14]. TM is the risk factor of VOD/SOS, and the incidence of VOD/SOS ranges from 6.1% to 33% in TM patients after HSCT [15–17]. Cappelli et al. have reported that oral defibrotide prophylaxis safely reduces the VOD/SOS incidence in pediatric thalassemic HSCT recipients [18]. Because defibrotide was not available in our transplant center, the prophylactic regimen for VOD/SOS in our study was a combination treatment of dalteparin and Lipo-PGE1. This regimen is commonly used in some centers for VOD/SOS prophylaxis although there is insufficient evidence to support the use of dalteparin and Lipo-PGE1 for VOD/SOS prophylaxis [19, 20]. In this study, the incidence of VOD/SOS was 10.4%. Such high incidence of VOD/SOS in our study might be attributed to pre-existing liver damage caused by iron overload, conditioning regimen with Bu and Cy, the use of CNIs for GvHD prophylaxis, and no defibrotide for VOD/SOS prophylaxis. These factors have been reported to be the risk factors for VOD/SOS.

The major risk factors have been identified as follows: elevated ferritin levels, unrelated HSCT, platelet refractoriness, sepsis post-HSCT, young age, conditioning regimens consisting of Bu and Cy, and the use of CNIs for GvHD prophylaxis [4]. In this study, moderate VOD/SOS was more frequently detected in MUD HSCT (8.7%) compared with MSD HSCT (3.1%) (*P* = 0.010). We found that the platelet engraftment was delayed, and the number of platelet transfusions was higher in VOD/SOS patients, while platelet refractoriness did not reach the significance for all-grade VOD/SOS (Tables 1 and 2). Unlike Cheuk et al., we found that there was no correlation between age and VOD/SOS [21]. Iron overload is prognostically important in HSCT, while we found that there was no correlation between the ferritin levels and VOD/SOS. Some studies have also shown that there is no correlation between the iron overload and VOD/SOS by using liver biopsy or liver magnetic resonance imaging (R2-MRI) to quantify liver iron content [21–23]. Our findings highlighted that it is necessary to perform prospective studies using direct measurements of iron overload rather than ferritin levels.

Most VOD/SOS fall into the mild or moderate category, and the proportion of severe VOD/SOS is about 25% [24]. Mild or moderate grade VOD/SOS is usually self-limited and reversible by supportive management. VOD/SOS is fatal in up to 50% of cases with severe VOD/SOS [24]. Initial reports of the use of defibrotide in the management of VOD show that the resolution rate of VOD is 36–76%, and the day +100 survival is 32–79% [25–29]. Richardson et al. have reported that earlier defibrotide treatment for VOD/SOS provides more favorable outcomes [30]. Initial reports of the use of methylprednisolone in the management of VOD indicate that the response rate of VOD is 63–67%, and the day +100 survival rate is 58–78% [31, 32]. However, defibrotide is not feasible in many transplantation centers. In our study, 59.3% of VOD/SOS patients were mild, 40.7% were moderate, and no patient developed severe VOD/SOS or died from VOD/SOS despite the lack of defibrotide in our center. We found that the 3-year OS rates and TFS rates of VOD/SOS patients were 94.3% and 92.5%, respectively. Our results were better compared with the historical reports, and the short-term withdrawal of CNIs after the early diagnosis of VOD/SOS might contribute to such a good result.

VOD/SOS arises from endothelial cell damage due to the transplantation conditioning regimen [4]. CNIs have damaging effects on the endothelium, leading to the aggravation of VOD/SOS [33]. Short-term withdrawal of CNIs after the diagnosis of VOD/SOS may prevent further deterioration of VOD/SOS. Of course, our results and interpretation should be cautious and need to be validated by further clinical research. On the other hand, the withdrawal of CNIs might increase the risk of GvHD. Our discontinuation of CNIs was short-term, and then CNIs were used again after the clinical symptoms of VOD/SOS were improved. In this study, methylprednisolone and basiliximab were administered to continue the prophylactic or therapeutic regimen of GvHD, which have been proved to be effective in the prophylactic and therapeutic regimen of GvHD [34–36]. In our study, the cumulative incidence of GvHD for patients with VOD/SOS was not significantly higher compared with patients without VOD/SOS, and all GvHD patients favorably responded to IST in this study. The 3-year GRFS rates for patients with or without VOD/SOS were 86.9% and 86.0%, respectively (*P* = 0.810). Our therapeutic measures could effectively and safely mitigate the mortality of VOD/SOS.

Collectively, our work showed that allo-HSCT was an effective approach for TM. The incidence of VOD/SOS was still high in TM patients after allo-HSCT. In the present study, we effectively managed VOD/SOS using an approach consisting of adequate supportive measures after the early diagnosis of VOD/SOS, short-term withdrawal of CNIs after the occurrence of VOD/SOS, and the use of methylprednisolone and basiliximab for GvHD prophylaxis. Taken together, this therapeutic strategy could benefit VOD/SOS patients.
